# The Sensorimotor Basis of Whisker-Guided Anteroposterior Object Localization in Head-Fixed Mice

**DOI:** 10.1016/j.cub.2019.07.068

**Published:** 2019-08-29

**Authors:** Jonathan Cheung, Phillip Maire, Jinho Kim, Jonathan Sy, Samuel Andrew Hires

**Affiliations:** 1 Department of Biological Sciences, Section of Neurobiology, University of Southern California, Los Angeles, CA 90089, USA; 2 Neuroscience Graduate Program, University of Southern California, Los Angeles, CA 90089, USA; 3 Lead Contact

## Abstract

Active tactile perception combines directed motion with sensory signals to generate mental representations of objects in space. Competing models exist for how mice use these signals to determine the precise location of objects along their face. We tested six of these models using behavioral manipulations and statistical learning in head-fixed mice. Trained mice used a whisker to locate a pole in a continuous range of locations along the anteroposterior axis. Mice discriminated locations to ≤0.5 mm (<2°) resolution. Their motor program was noisy, adaptive to touch, and directed to the rewarded range. This exploration produced several sets of sensorimotor features that could discriminate location. Integration of two features, touch count and whisking midpoint at touch, was the simplest model that explained behavior best. These results show how mice locate objects at hyperacute resolution using a learned motor strategy and minimal set of mentally accessible sensorimotor features.

## In Brief

Competing models exist for how mice use sensory and motor signals to determine the precise location of objects along their face with their whiskers. Cheung et al. test six of these models in head-fixed mice. The integration of two features, touch count and whisking midpoint at touch, is the simplest model that explains behavior best.

## Graphical Abstract

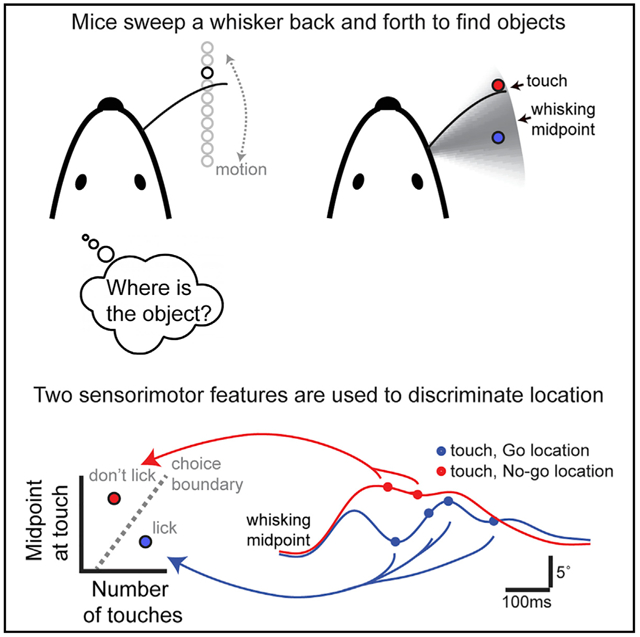

## INTRODUCTION

Locating objects through the sense of touch is an essential behavior across animal species. In humans and rodents, tactile object localization is an active process that combines directed sensor motion with mechanosensory signals. Rodents sweep their large whiskers back and forth and use the resulting tactile sensations to locate [1] and orient to objects [2] and guide navigation [3]. Identifying the motor strategies deployed and the resulting sensorimotor features that underlie object location perception during these behaviors is an essential step to understanding algorithms and neural circuit implementations of active sensory perception [4, 5].

Head-fixed preparations are advantageous for investigating object localization due to their exquisite level of experimental control, including the ability to monitor motion with high precision [6, 7]. Rodents can determine the location of objects by active exploration with whiskers [8], even when head fixed [9]. High-speed videography [10] and physical models [11-14] can quantify motion and forces that drive whisker input with submillisecond resolution during behavior [15, 16]. This input is transformed and integrated in a topographic arrangement of columns in primary somatosensory cortex (S1) that have a one-to-one mapping to individual whiskers. Examination of the activity patterns within and across these cortical columns has revealed how sensorimotor features of tactile exploration are represented and processed in the brain [17-21].

Whiskers project from an array of follicles arranged in columns and rows across the face. From posterior to anterior positions within a row, each large whisker (i.e., macrovibrissa) launches from its follicle at progressively greater azimuthal angles, with about 20° of angular difference between neighboring whiskers. Thus, discriminating object locations separated by ≥20° along the anteroposterior (i.e., horizontal) axis is trivial using multiple whiskers and a labeled-line code based on touch presence [22]. However, head-fixed mice with a full whisker field can do better than this and discriminate object location to at least 6° of resolution [9]. Achieving this hyperacute localization resolution is not trivial. Head-fixed mice can also discriminate well-separated anteroposterior locations (~15°) with a single whisker [23] using motor strategies that establish large differences in touch likelihood [24] or direction [25] between locations. The perceptual limits, motor strategies, and sensorimotor features that drive hyperacute object location perception remain unclear.

**Figure 1.**
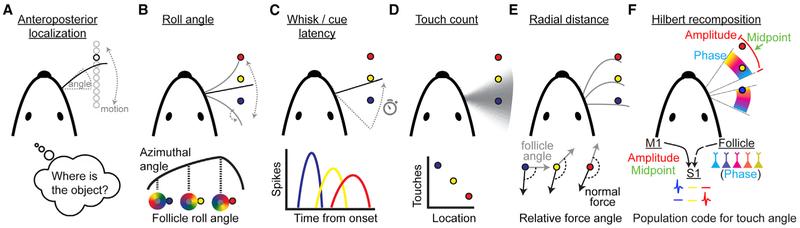
Models of Anteroposterior Object Localization (A) Schematic of task geometry. The whisker is actively swept back and forth to locate a pole (black circle). Angle is the azimuthal angle of the whisker at the follicle relative to the mediolateral axis of the animal. (B) Position is discriminated by how much the follicle has rotated at the moment of touch. (C) Position is discriminated by when spikes occur relative to onset of whisking. (D) Position is discriminated by the number of touches during a bout of directed exploration. (E) Position is discriminated by the degree to which the normal force of object is pointing laterally versus toward the follicle launch angle. (F) Position is discriminated by which neurons are activated by touch at specific angles. Angle is uniquely specified by the amplitude, midpoint, and phase of a whisk cycle. Activity from primary sensory neurons that are modulated by phase is combined with an internal representation of whisking amplitude and midpoint to activate distinct sets of neurons in S1 at the moment of touch, depending on the azimuthal angle at which touch occurs.

Several plausible models have been proposed for how rodents achieve location hyperacuity along the anteroposterior axis (Figure 1A), all of which have gaps in experimental support. These models differ in the sensorimotor features gathered and used to construct location perception. In a roll angle model (Figure 1B), rodents sense how much the whisker has rotated on its long axis at time of touch through a differing pattern of mechanoreceptor activation [22, 26]. In a whisk latency model (Figure 1C), rodents measure the time of touch referenced to the time from maximum retraction. More anterior objects take longer to reach [27]. Similarly, in a cue latency model (Figure 1C), mice measure the time from cue-triggered whisking onset to the time of touch across one or more whisks. In a touch count model (Figure 1D), rodents direct their whisking to a location range, so objects more central to this range generate more touches and consequently more spikes in S1. Location is read out by spike count in S1 with more central objects represented by more spikes [24, 28]. In a radial distance model (Figure 1E), rodents measure the distance between follicle and object by comparing the angle of the normal force relative to the angle of the follicle. This varies depending on where along the whisker touch occurs due to increasing whisker flexibility with distance [29-32]. In a Hilbert recomposition model (Figure 1F), rodents integrate three Hilbert components of whisker motion (amplitude, midpoint, and phase) to compute the azimuthal angle of the whisker at time of touch [33]. Amplitude and midpoint originate from primary motor cortex (M1) as efference copy [34], and phase is encoded in a reafferent sensory signal from the whisker follicle [35-37]. Despite this proliferation of models, no consensus has emerged for which approach, or mix thereof, is actually used.

Here, we use behavioral manipulations and statistical learning to identify the simplest model that best explains localization hyperacuity with a single whisker. We quantify the perceptual limits of anteroposterior localization and determine what motor strategies are deployed, how they influence sensorimotor information gathered, and how this information influences location perception. We identify a two-stage classifier that combines touch count with whisking midpoint at touch as the simplest, best performing model. This provides insight into where and how active location computations are performed by neural circuits and a foundation from which natural object localization can be better understood.

## RESULTS

### Task Design and Animal Performance

**Figure 2.**
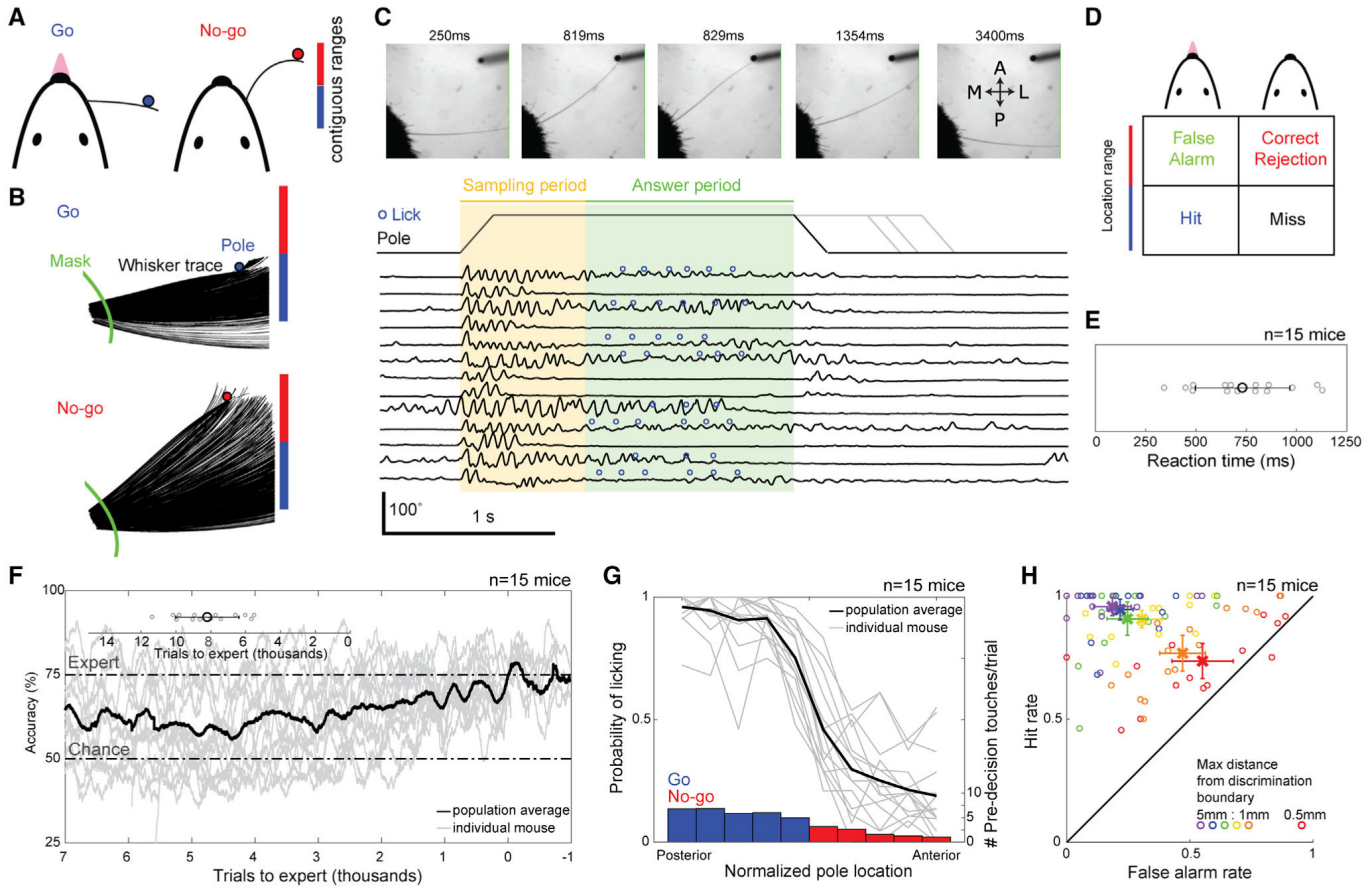
Head-Fixed Task and Performance (A) Trained mice report the perceived location of a pole presented along the anteroposterior axis via licking (go) or not licking (no-go). (B) Overhead view of tracked whisker for two trials. To eliminate variation from fur, azimuthal angle is determined at the intersection of mask and whisker trace. (C) Trial structure with example imaging frames at top. Pole presentation is triggered 500 ms from session start and takes ~200 ms to come into reach. Azimuthal angle time series for 15 consecutive trials is overlaid with the sampling period (750-ms duration), answer period (1,250-ms duration), and licks. (D) Possible trial outcomes based on pole presentation and mouse choice. (E) The average reaction time for each individual mouse (gray circles) and the mean ± SEM for all mice (black circle). (F) Learning rates for this task highlighting 7,000 trials before and 1,000 trials after reaching expert (75% accuracy over 200 trials). Inset, number of trials required to reach expert for each mouse in gray and population in black (mean ± SD; 8,194 ± 1,816 trials). (G) Psychometric performance curves for individual mice (gray) and across the population (black) expert in the task (n = 15 mice). Bars denote the mean number of touches prior to decision for go (blue) and no-go (red) trials. (H) Performance between go/no-go pairs of bins with the max distance of 0.5, 1, 2, 3, 4, and 5 mm from the discrimination boundary. Circles denote individual mice. X denotes mean ± SEM across the population. p values comparing population hit trials to false alarm trials are as follows: 0.5 mm p = 9.2e–3; 1 mm p = 5.2e–13; 2 mm p = 1.5e–15; 3 mm p = 1.5e–15; 4 mm p = 1.5e–15; and 5 mm p = 1.5e–15; 2-sample t-test (t-stat, degrees of freedom: 0.5 mm = 2.6, 276; 1 mm = 7.4, 552; 2 mm = 20.0, 596; 3 mm = 21.8, 569; 4 mm = 26.4, 578; 5 mm = 31.8, 594).

To investigate the sensorimotor basis of object location perception, we used a variation of a go/no-go whisker-guided localization task in head-fixed mice [9] that requires precise knowledge of object location to achieve maximum performance. We trained water-restricted head-fixed mice (n = 15) to discriminate the location of a smooth vertical pole randomly presented in contiguous ranges of go (0–5 mm) and no-go (5–10 mm) positions along the anteroposterior axis of the animal, about 8 mm lateral from the whisker pad (Figure 2A). Mice were trimmed to a single whisker at the start of training, maintained until task mastery. We traced whisker motion, touch, and deflection from an overhead view at 1,000 fps (Figure 2B).

For each trial, the pole was presented for at least 2 s in the go range (50% trials) or no-go range (50% trials). The pole moved vertically into and out of the field with the onset of motion associated with a 250 ms sound of a pneumatic valve, which cued mice to task structure. Mice voluntarily explored the pole and their surroundings with their whisker (i.e., whisking) during the sampling period (0.75 s duration) and reported their perception of object location by licking or not licking during the answer period (1.25 s duration; Figure 2C). The response choice led to different trial outcomes based on pole location (Figure 2D). On hit trials, mice were rewarded with a water droplet (4–8 μL), and on false alarm trials, mice were punished with a 2 s timeout. Correct rejection and miss trials were neither rewarded nor punished. Licking extended the duration of the pole presentation.

In all analyses, we only consider sensorimotor behavior (e.g., whisks and touches) that contributed to a decision by including only data before the decision lick. The decision lick is defined as the first lick in the answer period or, on no-lick trials, the median of the decision lick times. This cutoff excludes post-decision motor activity that is driven by rhythmic licking on hit and false alarm trials. The reaction time between the first touch in a trial and the decision lick was 736 ± 240 ms (741 ± 249 ms on hit trials and 690 ± 243 ms on false alarm trials; Figure 2E). Whisker motion resulted in a change of azimuthal angle (i.e., angle) of the whisker base relative to the mediolateral axis of the mouse. Across all mice, this angle at onset of touch (i.e., touch angle) spanned 49.4° ± 8.8° from extreme posterior to anterior pole positions. Touch angle for the go and no-go range varied across sessions and was affected by the radial distance of the pole presentation axis and translation of the follicle during whisking. Across all touched pole positions, the follicle translated by 1.5 ± 0.3 mm total (1.3 ± 0.4 mm in the anteroposterior axis and 0.7 ± 0.1 mm in the mediolateral axis). Mice performed 485 ± 179 trials per session. It took 8,194 ± 1,816 trials (Figure 2F; excluding one outlier of 19,923 trials) to reach expert performance, defined as >75% accuracy over 200 trials.

To determine the spatial precision of localization, we examined trials near the go/no-go discrimination boundary. On average, there was a significant change in lick probability between go and no-go trials when the pole was presented ≤1 mm (3.8° ± 0.5° mean angle difference; 29% ± 11% lick difference; p = 4.4e–5; two-tailed t test) or ≤0.5 mm (1.9° ± 0.4° mean angle difference; 18% ± 20% lick difference; p = 0.03; two-tailed t test) from the boundary (Figures 2G and 2H). This indicates mice can discriminate above chance with submillimeter precision along the anteroposterior axis with a single whisker. The mean number of pre-decision touches per trial (i.e., touch count) decreased from most posterior (6.8 ± 2.6) to anterior bin (1.1 ± 0.9; Figure 2G).

### Behavior Is Consistent with Closed-Loop Integration of Sensorimotor Cues for Hyperacute Object Localization

**Figure 3.**
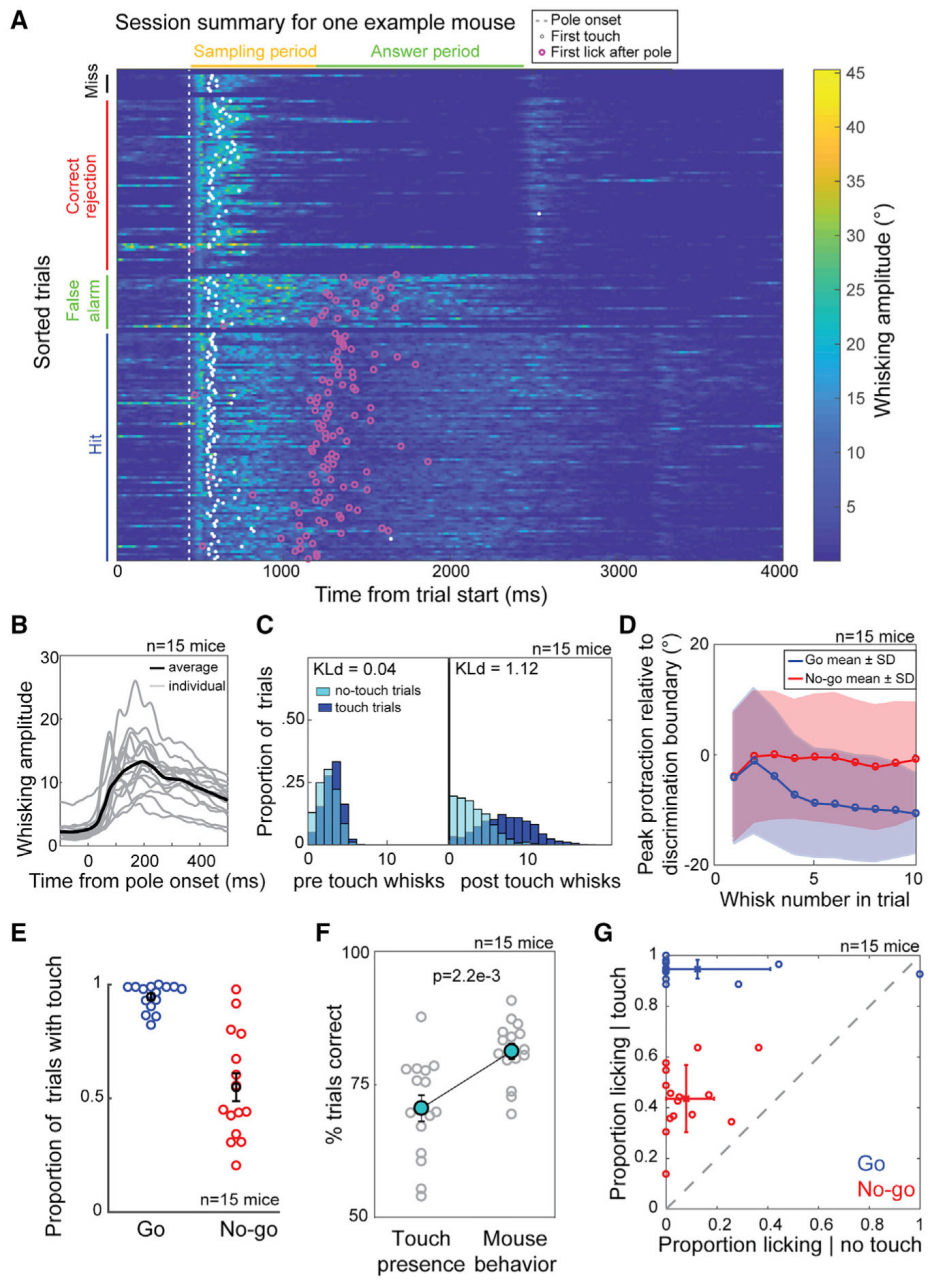
Motor Strategy and Its Influence on Patterns of Touch (A) Heatmap of whisking amplitude for one mouse. Trials are sorted with first at the bottom and grouped by trial outcome. White dots are time points of first touch. Magenta circles show time points of first lick after onset of pole presentation. (B) Whisking amplitude relative to time of pole onset for each mouse (gray) and average for all mice (black). Mean ± SD of whisking onset from cue is 60 ± 16 ms. (C) Left: population distribution for the number of whisks before first touch. Right: population distribution of the number of whisks after first touch and before decision is shown. For no-touch trials, the median first touch time for that mouse was used. Distribution difference is quantified using Kullback-Leibler divergence (STAR Methods). (D) Mean ± SD of the peak protraction relative to the discrimination boundary for each whisk in a go (blue) or no-go (red) trial before decision. (E) Proportion of trials with touch for each mouse based on trial type. Bars represent SEM. (F) Trial type prediction performance of a logistic classifier based on touch presence compared to each mouse’s trial type discrimination performance. Bars represent SEM. p = 2.2e–3; Wilcoxon signed-rank test. (G) The proportion of go or no-go trials in which licking occurs conditioned on whether touch occurred on that trial. Bars represent SD.

Active exploration during object localization was intentional, adaptive, directed, and noisy. Mice initiated whisking in a stereotyped manner throughout a session, regardless of trial outcome (Figure 3A). Mice held their whiskers steady outside of the period of pole availability and began vigorously whisking to the sound of pole-in with short latency (60 ± 16 ms; Figure 3B). This shows that active exploration for object localization is an intentional process triggered by a cue.

Mice made 2.5 ± 1.2 whisks before the first touch on trials with touch and 1.9 ± 1.2 whisks before the median time of those first touches on trials without touch. There was no significant difference in the two distributions (Kld 0.04; Figure 3C), which shows that failure to touch on a trial is not due to a failure to initiate a motor program. However, mice made many more whisks (6.5 ± 3.2) after the first touch on trials with touch, compared to the number of whisks (2.5 ± 2.1; Kld 1.12; Figure 3C) after the median time of those first touches on trials without touch. This demonstrates that mice deploy an exploration strategy that is adaptive to sensory feedback, consistent with a closed-loop model of tactile perception [21, 38, 39].

We quantified the motor strategy and its precision by the angle of maximum protraction for each whisk cycle. The first two whisks on go and no-go trials were targeted to the discrimination boundary (Figure 3D). On these whisks, the pole was generally still ascending and not yet in reach. From the third whisk, the go and no-go trials diverged. On go trials, the peak protraction settled around 10° posterior to the decision boundary, due to physical restriction by the pole (Figure 3D). On no-go trials, average peak protraction was maintained at the discrimination boundary. If this average motor strategy was executed with no variance, it would result in at least one touch for go positions and zero touches for no-go positions on each trial, essentially transforming our precise discrimination task into an active detection task. However, the whisk-to-whisk variance was large (10.9° mean SD), suggesting a noisy execution of the motor plan. This variance resulted in mice touching the pole on 94.6% ± 1.5% SEM go trials and 54.9% ± 6.1% SEM no-go trials (Figure 3E). A logistic classifier based on the presence or absence of touch discriminated go from no-go locations with 70.5% ± 9.5% accuracy, but mice significantly outperformed this, correctly discriminating 81.2% ± 5.7% of trials (Figure 3F). Furthermore, mice licked on 94.7% ± 3.6% of go trials with at least one touch (i.e., touch trials) but only licked on 43.6% ± 13.2% of no-go touch trials (Figure 3G).

These data show that mice direct their exploration to produce a difference in probability of touch between go and no-go positions. If they fail to touch, they rarely lick, similar to a detection task. However, if they do touch, they still are able to discriminate the object location. Thus, they must be interpreting additional sensorimotor features of touch to locate the object.

### Sensorimotor Features at Touch that Discriminate Location and Choice

**Figure 4.**
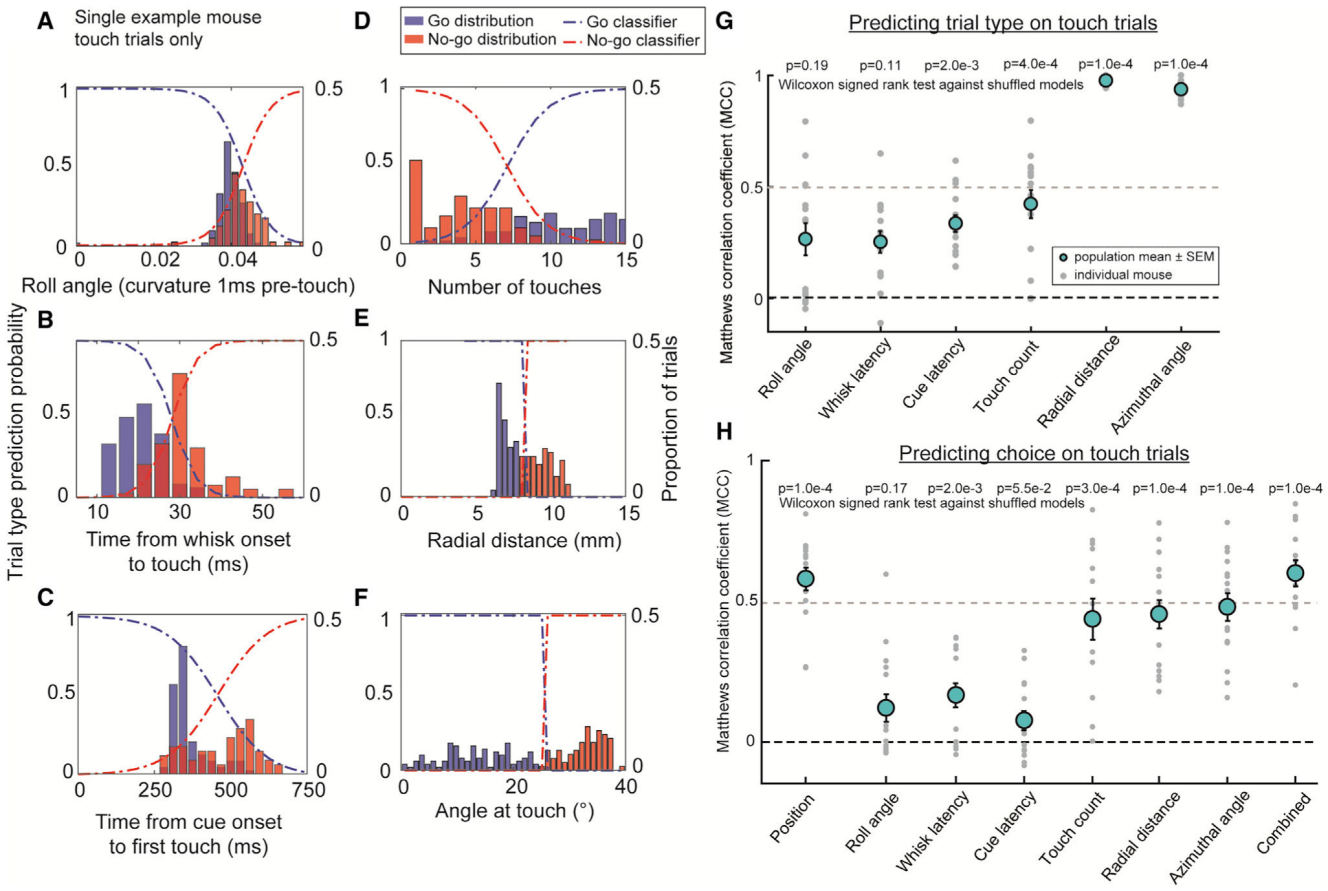
The Distribution of Sensorimotor Features and Their Utility for Predicting Trial Type and Choice (A–F) Histogram of feature values and classifiers for predicting trial type using (A) roll angle, (B) whisk latency, (C) cue latency, (D) touch count, (E) radial distance at touch, and (F) angle at touch. (G) Trial type prediction performance of logistic classifiers for all mice based on each of the six features. Bars represent SEM. p values; Wilcoxon signed-rank test against shuffled models. Touch trials only are shown. See also Figure S1. (H) Choice prediction performance of logistic classifiers for all mice trained on pole position, each of the six features, or all six features combined. Bars represent SEM. Touch trials only are shown. values; Wilcoxon signed-rank test against shuffled models. See also Figure S2.

What features of touch could mice *possibly* use to discriminate location? We examined how six sensorimotor features associated with proposed hyperacute localization models (Figure 1) were distributed at the instant of touch. The torsional roll angle, quantified by the apparent whisker curvature 1 ms prior to touch [22], was greater for no-go versus go locations (Figure 4A). More posterior locations had shorter average time from whisk onset to touch in each whisk cycle (Figure 4B). Similarly, go positions were associated with shorter latency from cue to first touch, because mice tended to need fewer whisks before hitting the pole (Figure 4C). There were more touches on go trials than no-go trials (Figure 4D). The radial distance from follicle to pole was greater for no-go trials (Figure 4E). The azimuthal angle at touch was more protracted on no-go trials (Figure 4F).

Using supervised learning, we built a logistic classifier to identify trial type using each of the above features on touch trials. By definition, touch presence had no discrimination power on these trials and anteroposterior pole location discriminated perfectly. We quantify performance using Matthew’s correlation coefficient (MCC) to account for the unbalanced distribution of touch trials between go and no-go positions in the training set (Figures S1A and S1B; STAR Methods). Unsurprisingly, radial distance (MCC 0.98 ± 0.02; accuracy 98.9% ± 0.1%) and azimuthal angle at touch (MCC 0.93 ± 0.04; accuracy 97.0% ± 1.4%) were the best discriminators, because they had the least overlap and were dependent on the task geometry rather than behavior. Roll angle (MCC 0.23 ± 0.26; accuracy 71.3% ± 9.4%) and whisk latency (MCC 0.25 ± 0.19; accuracy 71.4% ± 5.9%) were the worst predictors. Cue latency (MCC 0.33 ± 0.15; accuracy 73.1% ± 6.9%) and touch count (MCC 0.43 ± 0.07; accuracy 77.4% ± 2.1%) were significantly better than chance (Figures 4G and S1C).

What features of touch do mice *actually* use to discriminate location? We built a logistic classifier to predict choice using each of the above features. Two models based on actual pole position or all other features combined were reference standards. Touch count, radial distance, and azimuthal angle classifiers predicted choice best, significantly better than shuffled models (Figure 4H). Due to the multicollinearity of features, a combined classifier with mean normalized features and L1 regularization allowed us to determine which features were most predictive in each mouse (STAR Methods). In the combined classifier, the average feature weight of touch count, distance, and angle were significantly different from zero (Figure S2). This supports that touch count and correlates of radial distance or azimuthal angle at touch, but not roll angle or timing based cues, are used to refine choice on touch trials.

### Identifying the Simplest Model that Predicts Choice Best

Having narrowed down potential sensorimotor drivers of choice, we sought to find the simplest set of features that predicted choice best. Touch count was the only choice-predictive feature under active control of the mouse (distance and touch angle are primarily dependent on geometry in this task). Therefore, we reasoned that mice may exclusively use touch count to drive choice.

**Figure 5.**
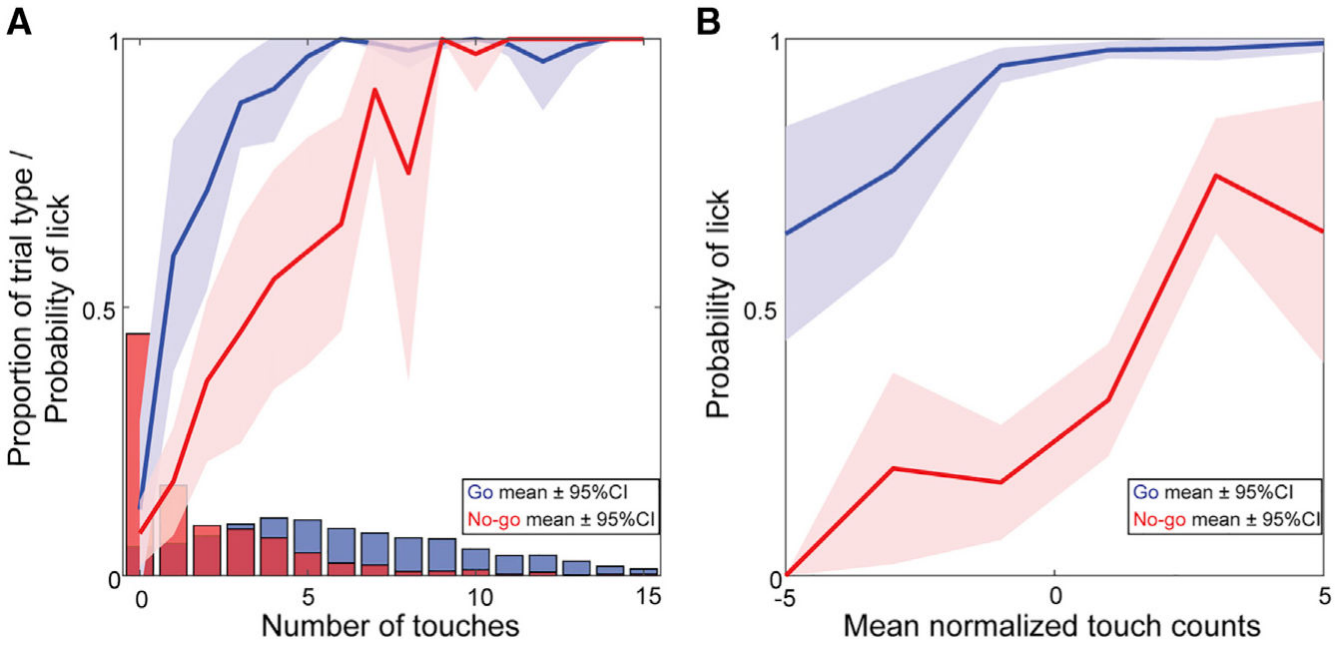
Mice Discriminate Location Using More Than Touch Count (A) Population average of touch count distributions and associated lick probabilities for all mice in go (blue) and no-go (red) trials. p values for 0 to 5 touches = 0.64, 4.4e–4, 1.7e–3, 1.1e–4, 7.0e–4, 5.8e–3; two-tailed paired t test (t-stat, degrees of freedom: 0 touches = 0.48, 13; 1 touch = 4.9, 11; 2 touches = 3.9, 13; 3 touches = 5.3, 4; 4 touches = 4.4, 13; 5 touches = 3.5, 10). (B) Touch count influence on licking controlled for pole position. Number of touches normalized to mean number of touches for each pole position plotted against lick probabilities for go (blue) and no-go (red) trials. Lick probabilities are shown as mean ± 95% confidence intervals.

If touch count was the only feature that drove choice, then mice should lick at equal probability on go and no-go trials that have identical numbers of touches. This was not the case. Reminiscent of the results on touch presence (Figure 3G), mice were significantly more likely to lick on go than on no-go trials with equal touch counts (Figure 5A). Surprisingly, greater stimulus sampling (i.e., more touches) decreased the difference in lick probability between trials with equal touches. Because touch count varies with pole position (Figure 2G), we isolated the effect of touch count on choice by comparing the difference between actual and average number of touch counts for that pole position. Trials with a higher number of touches than usual for that position had higher lick probability (Figure 5B), particularly on no-go trials. This shows that touch count has a direct effect on choice, but additional features also must be used.

**Figure 6.**
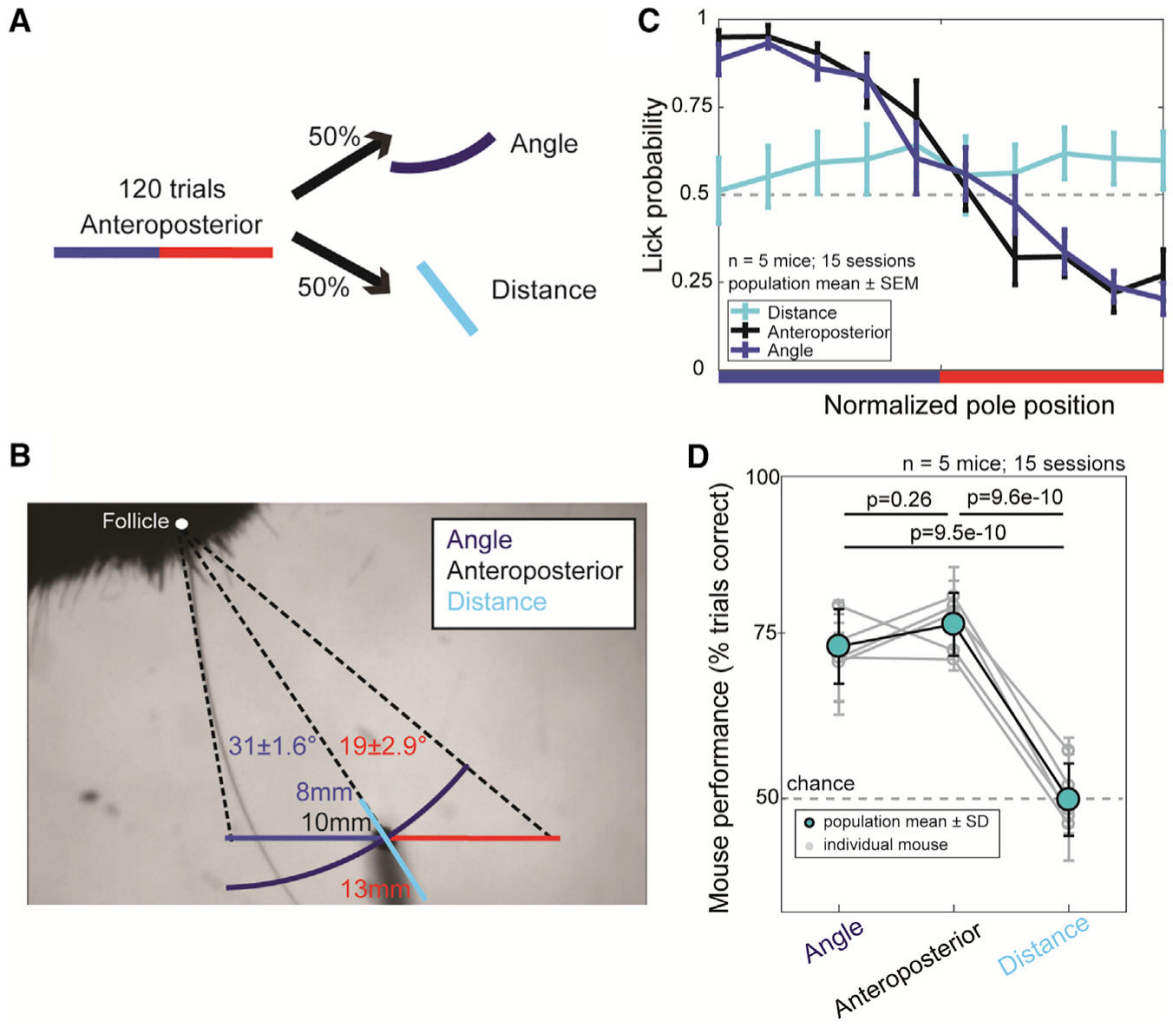
Mice Discriminate Location Using Features Correlated to Azimuthal Angle Rather Than Radial Distance (A) Task design. After 120 trials of anteroposterior pole presentation, angle or distance trials were presented with 50% probability. (B) The angle presentation positions (blue) held distance to the discrimination boundary constant while varying azimuthal angle across the anteroposterior task range. The distance presentation positions (cyan) held azimuthal angle fixed to the discrimination boundary angle while varying distance across the anteroposterior task range. Go positions spanned a range of 31° ± 1.6° or 8–10 mm distance, and no-go positions spanned 19° ± 2.9° or 10–13 mm distance. (C) Mean psychometric performance curves ± SEM for each class of trials across the population (n = 5 mice, 15 sessions). (D) The mean performance for angle trials was not significantly different from anteroposterior trials (p = 0.26; one-way ANOVA). Distance trials performance was at chance and significantly different from the anteroposterior and angle task (anteroposterior p = 9.6e–10, angle p = 9.5e–10; one-way ANOVA [F-value, degrees of freedom = 96.5, 36]). Bars represent SD.

The two remaining choice-predictive features, radial distance and touch angle, are tightly correlated in this anteroposterior localization task. To disentangle their influence on choice, we introduced a task variation that decomposed the discrimination to solely depend on distance or angle. To establish the baseline context, five expert mice were first presented 120 trials of the anteroposterior task. Then they were presented with randomly interleaved distance and angle trials (Figure 6A). Distance trials matched the contiguous distribution of radial distance while holding azimuthal angle fixed at the value of the anteroposterior discrimination boundary. Angle trials were vice versa (Figure 6B). Psychometric performance curves for angle trials were indistinguishable from anteroposterior trials in all mice (n = 5 mice, 15 sessions), and performance fell to chance levels on distance trials, with a small bias toward licking (Figures 6C and 6D). This demonstrates that mice do not use distance to the pole to achieve anteroposterior location hyperacuity. Instead, they use features that co-vary with the azimuthal position of the pole.

**Figure 7.**
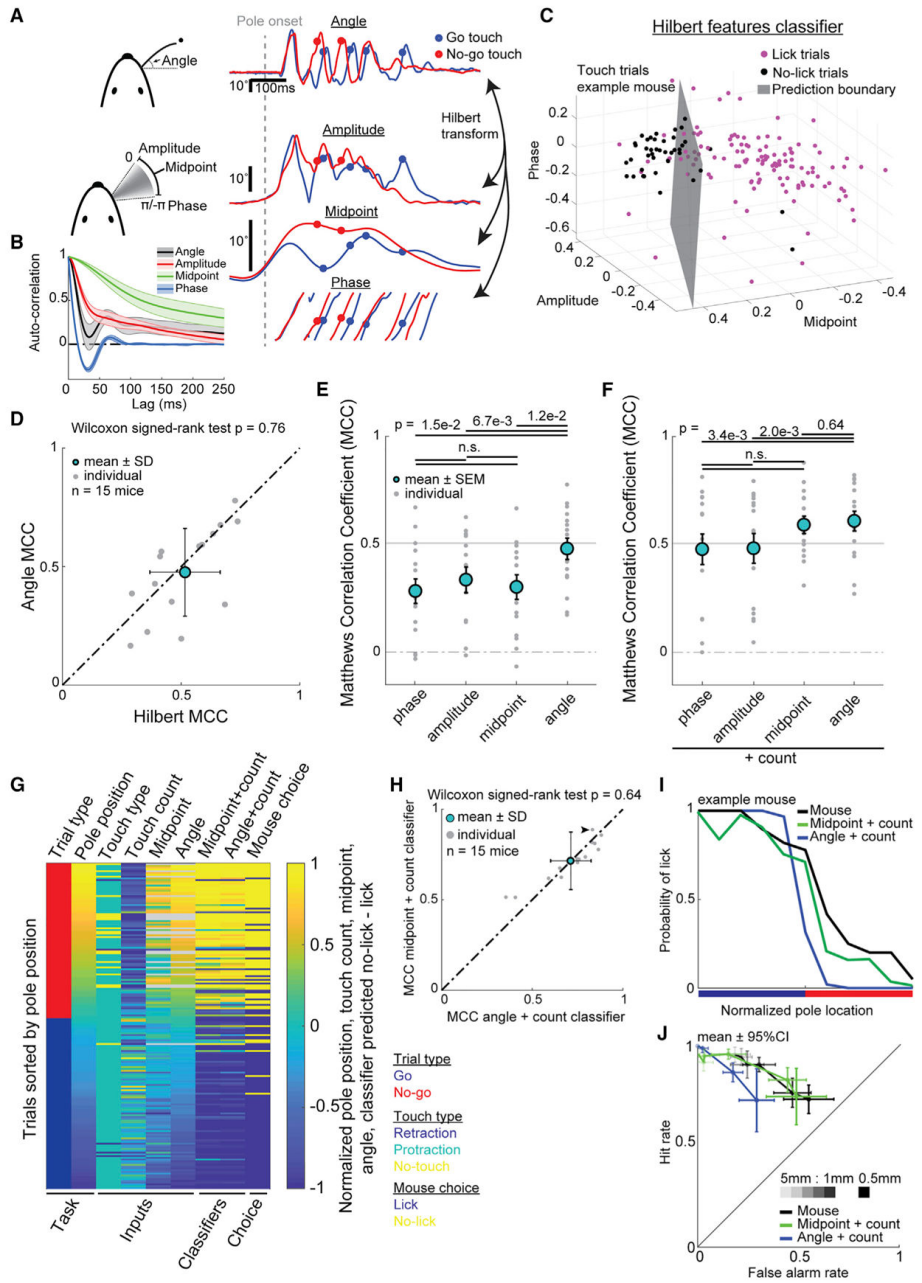
Choice Can Be Best Predicted by a Combination of Touch Count and Whisking Midpoint at Touch (A) Time-varying azimuthal angle can be transformed to the Hilbert components amplitude, midpoint, and phase. Example exploration bout for go (blue) and no-go (red) trial is shown. (B) Average autocorrelation across all mice for angle, amplitude, midpoint, and phase. (C) Choice prediction space for one mouse using Hilbert features. (D) Classifier performance measured using MCC between angle and Hilbert features. Bars represent SEM. p = 0.76; Wilcoxon signed-rank test. (E) Performance (MCC) of classifiers trained with individual model components versus angle at touch. Bars represent SEM. Significant differences: angle to phase (p = 1.5e–2); amplitude (p = 6.7e–3); and midpoint (p = 1.2e–2). Non-significant differences: phase to amplitude (p = 0.23); phase to midpoint (p = 0.80); and amplitude to midpoint (p = 0.52). All compared using Wilcoxon signed-rank test. (F) Performance (MCC) of classifiers trained with individual model components plus touch count versus angle at touch plus touch count. Bars represent SEM. Significant differences: angle to phase (p = 3.4e–3) and amplitude (p = 2.0e–3). Non-significant differences: phase to amplitude (p = 0.64); phase to midpoint (p = 19); amplitude to midpoint (p = 0.12); and angle to midpoint (p = 0.64). All compared using Wilcoxon signed-rank test. (G) Heatmap of one sorted session task structure, sensorimotor inputs, classifier predictions, and mouse choice. Continuous variables (pole position, touch count, midpoint at touch, angle at touch, midpoint + touch count choice prediction, and angle + touch count choice prediction) are normalized from minimum (−1) to maximum (+1). NaN data are gray. Categorical variables (trial type, primary touch direction, and mouse choice) are colored as in the legend. See also Figure S3. (H) Comparison of midpoint + touch count and angle + touch count classifiers for all trials. Bars represent SEM. p = 0.64; Wilcoxon signed-rank test. Black arrow denotes mouse shown in example in (G). See also Figure S4. (I) Psychometric curves for optimal trial type discrimination performance using midpoint + counts and angle + counts compared against mouse choice for example mouse in (G). See also Figure S5. (J) Comparison of discrimination resolution between optimal trial type classifiers and mouse performance from Figure 2H. Shading denotes distance from discrimination boundary.

This leaves the perplexing question of how azimuthal angle at touch can influence choice, because the azimuthal angle of the whisker is not directly encoded by primary sensory afferents [40-45]. An influential model has been proposed for how the brain could compute this angle from mentally accessible time-varying features. Whisker angle motion can be losslessly transformed into three components, amplitude, midpoint, and phase, using the Hilbert transform (Figure 7A). These components vary in their characteristic timescales, with phase changing completely during a single whisk cycle and midpoint remaining most similar across multiple whisk cycles (Figure 7B). Neural correlates of amplitude and midpoint are found in areas of M1 that project to S1 [34], and correlates of phase are found in ascending projections from the follicle to S1 [35]. The Hilbert recomposition model supposes that these components of time-varying angle are combined with touch time to produce a precise, unambiguous representation of azimuthal angle at touch [33] (Figure 1F). We tested whether the Hilbert recomposition model is consistent with behavior by training a choice classifier on the average of each of these three components for all pre-decision touches in each trial (Figure 7C). To avoid difficulties associated with fitting a periodic variable, phase, with a logistic function, we only considered trials that had protraction touches (89.9% ± 5.9% SD of the touch trials). This Hilbert recomposition classifier performed similarly (MCC 0.51 ± 0.17) to angle at touch (MCC 0.48 ± 0.19; Figure 7D). Thus, this model, although complex, is a plausible means of computing whisker angle to construct location perception.

To assess whether a simpler model could explain choice equally well on trials with touch, we trained choice classifiers on only one Hilbert component at a time. On average, these classifiers performed similarly to each other and worse than angle or touch count alone (Figure 7E). Which classifier performed best varied between mice. To determine whether touch count provided redundant or complementary information about choice, we tested the combination of each component with touch count on touch trials (Figure 7F). Adding touch count improved each classifier’s performance. Midpoint + touch count achieved the highest average performance (MCC 0.59 ± 0.04 SEM). This was indistinguishable from the performance of angle + touch count (MCC 0.61 ± 0.05 SEM). Angle + touch count performance remained significantly better than phase or amplitude + touch count (MCC 0.48 ± 0.07 SEM; MCC 0.48 ± 0.07 SEM). Thus, touch count provides complementary location information which, when combined with one Hilbert component, whisking midpoint at touch, predicts mouse choice as well as models that compute or use the exact touch angle.

Although these classifiers predicted choice well for trials with protraction touch, some trials have no touches or exclusively retraction touches. To fully assess classifier performance, we applied the same classifiers to all trials. Because sensorimotor features at touch are undefined on trials without touch, the classifiers were implemented in two steps. For trials without touch, choice was predicted using touch count alone, which invariably predicted “no lick” for those trials. For trials with touch, either angle or midpoint at touch was combined with touch count to predict choice (Figures 7G and S3). These two classifiers predicted choice equally well (midpoint + count MCC 0.71 ± 0.03 SEM, accuracy 87.2% ± 3.9%; angle + count 0.72 ± 0.04 SEM, accuracy 87.5% ± 4.7%) with little difference in performance between mice (MCC r^2^ = 0.87; Figure 7H) and essentially the same performance as the protraction touch only trials. Amplitude or phase with touch count also performed reasonably well, though significantly worse than angle with touch count (Figure S4).

To determine whether midpoint and touch count provide sufficient information about pole location to account for mouse performance, we trained trial type classifiers on either midpoint + touch count or angle + touch count and compared their predictions to psychometric performance curves of mice. We found that both classifiers provide sufficient information about pole position, but the midpoint + touch count classifier better fit the psychometric curves in 14/15 mice (Figures 7I and S5). Furthermore, the discrimination resolution of midpoint + touch count was a better match to mouse performance than angle + touch count (Figure 7J). Together, these data best support a simple model of active tactile perception, where mice deploy targeted, noisy, adaptive exploration and use their sense of touch count combined with whisking midpoint to locate objects with submillimeter precision.

## DISCUSSION

We assessed how well the sensorimotor features associated with six models of active tactile perception (Figure 1) could discriminate object position and predict choice during head-fixed anteroposterior object localization. Mice achieved hyperacuity with a single whisker, discriminating locations separated by ≤0.5 mm and <2° (Figure 2). The directed and adaptive search strategy used by mice (Figure 3) made the number and characteristics of touches to be predictive of object location and choice (Figure 4). Mice discriminated location on trials with equal numbers of touches, suggesting location perception was refined by other sensorimotor features when touch occurred (Figure 5). By independently manipulating the distance or angle of the presented object during localization, we found that azimuthal angle, but not radial distance, also drove choice (Figure 6). A model for computing azimuthal angle from three Hilbert components of whisker motion predicted choice as well as angle on touch trials (Figure 7). When combined with touch count, a single Hilbert component, midpoint, predicted choice as well as azimuthal angle with touch count, showing that computing azimuthal angle is not necessary (Figure 7). This supports a model where neural correlates of touch count and a single motor feature, midpoint, are integrated to produce hyperacute perception of object location along the anteroposterior axis of the mouse face.

We note several limitations of our work. We relied on a single overhead view of whisker curvature to estimate torsional roll angle, which is subject to greater measurement noise than a 3D reconstruction from multiple camera angles. We also did not account for inertial bending during whisking. Despite these caveats, a prior study showed a tight linear relationship between whisker curvature in an overhead projection and roll angle in this range of protraction angles in rats [22], which supports that curvature is a good enough proxy for roll angle here.

Although azimuthal angle at touch nearly perfectly predicted pole location, the best classifiers only achieved 87% performance in predicting choice. This remaining unpredictable variability may reflect internal changes in motivation, attention, satiety, or frustration that were uncontrolled. Pupillometry and facial expression tracking may allow a more precise accounting for the role of internal state changes on choice in future work. Alternatively, reward or choice history may influence choice during these types of tasks. A recent study of whisker-based object localization found that choice history had a significant influence on error trials [25]. However, we were unable to find an influence of choice history on choice in our current study. This may be because we restricted analysis to expert mice in a contiguous block of 200 trials in the middle of each session, where motor engagement and performance was high and stable.

The insights this work provides about how mice naturally explore the world are necessarily limited by our experimental constraints: the mice are trimmed to a single whisker, head-fixed, and highly trained within a stable environmental context. In freely moving mice, the initial strikes of an object along a side of the face are likely to be with a single whisker due to a number of factors. Many whiskers are often missing from cohabitating mice, due to trimming associated with social hierarchy in C57/BL6 mice [46]. The progressive length of whiskers across the pad [13] prevents short whiskers from reaching distant objects. Rodents use tactile feedback to adapt their whisking pattern, minimizing impingement during object contact [47]. Initial whisker contacts are followed by a shift to asymmetric whisking attempting to bring more macrovibrissae into contact and an orienting head movement to explore more carefully with their microvibrissae [48]. Head-fixed tasks are thus relevant to the localization computations that guide contact-induced whisking asymmetry and head orientation to objects.

Classifiers based on roll angle, whisk latency, or cue latency each performed relatively poorly at discriminating location and predicting choice. Although mice roll their whiskers through cycles of protraction and retraction, our results suggest the variance in this rotation is too great to be useful for precise location discrimination. Experimental support of whisk latency models are primarily based on electrically evoked artificial whisking in anesthetized rats [27], which has minimal trial-to-trial variance in whisker motion. Our results suggest that it is difficult to use timing of touch referenced to a point of the whisk cycle for precise location discrimination during active whisking due to the variability in amplitude, midpoint, and velocity across whisking cycles [41]. Likewise, cue latency requires less whisking variance to be useful for precise location discrimination. On the other hand, classifiers based directly on angle or angle computed from Hilbert components discriminated location and predicted choice well. Adding touch count as a feature improved choice prediction, showing its importance for driving choice. Because choice classifiers trained on midpoint + touch count equaled the performance of angle + touch count (Figures 7F-7H), we conclude that mental computation of the exact angle of the whisker at the exact time of touch [33] is unnecessary to precisely locate objects.

A pure touch count model is supported by prior work showing that the number optogenetic stimulation pulses applied to layer 4 (L4) of S1, but not their millisecond precise timing, influences illusory perception of object location [24]. Yet we show that mice discriminate location when identical numbers of touches occur (Figure 5). In the same prior study, stimulation needed to be coincident with whisking to influence perception. This suggests that touch signals may be referenced to a slowly changing motor variable to refine location perception during active exploration. The use of midpoint, which is auto-correlated across whisk cycles (Figure 7B), may provide mice with a way to average out the variability in whisking during bouts of exploration. Because trial type classifiers trained on midpoint + touch count matched the discrimination performance of mice better than angle + touch count (Figures 7I and 7J), we conclude that whisking midpoint is more likely to be used than angle to precisely locate objects.

Perhaps our most surprising observation was that mice had a higher false alarm rate when they made more touches (Figure 5). This was contrary to our expectation that those trials would either show higher performance, because pole position would be sampled more times, or at least the same performance, as in a decision-making model where evidence is accumulated until a confidence boundary is reached. Rats performing active texture discrimination also vary the number of touches prior to a decision, but their performance is independent of touch count [39], consistent with bounded evidence accumulation seen in tactile [21], auditory [49], and visual discrimination [50] tasks.

A key difference between active object localization and these tasks explains our surprising observation. In texture discrimination, mice must touch the object to gather evidence. In active localization, both the presence and absence of touch provide evidence of object location. Directed exploration makes the act of touching, not just the properties of the touch, a location informative feature. This distinction also explains why mice increase their motor engagement if touch occurs (Figure 3C). More whisking causes more touches, but noisy, directed whisking (Figure 3D) makes the chance of touching less for no-go positions than for go positions. Thus, touch count per se informs the mouse’s choice, and the closed-loop motor response to touch tends to increase the separation in distributions of this decision-informative feature between go and no-go trials.

Our finding that midpoint and touch count together best predict choice in this task has implications for the origin and site of integration of sensorimotor signals that drive location perception. Whisking midpoint is correlated to neural activity in M1 [34], reflects the relative activity of intrinsic and extrinsic muscles in the whisker pad [51], and changes over timescales of hundreds of milliseconds (Figure 7B). M1 axons strongly excite the tuft and proximal dendrites of thick-tufted L5B neurons in S1 [52]. Calcium responses in the axons of these projection neurons [53] and the dendritic tuft of L5B-recipient neurons in S1 correlate to object location [54]. Activity of these L5B neurons forms a distributed representation of object location [55]. Meanwhile, touch count is tightly correlated to the spike count in L4 excitatory neurons of the primary whisker barrel in S1 [28]. These L4 S1 neurons strongly excite L5B proximal dendrites of S1 [56]. Combining this evidence with our new behavioral results suggests L5B neurons in S1 as a prime candidate for where midpoint and touch count signals are integrated to drive perception of object location.

## STAR★METHODS

### LEAD CONTACT AND MATERIALS AVAILABILITY

Further information and requests for resources and reagents should be directed to and will be fulfilled by the Lead Contact, Samuel Andrew Hires (shires@usc.edu). This study did not generate new unique reagents or mouse lines.

### EXPERIMENTAL MODEL AND SUBJECT DETAILS

Fifteen VGAT/ChR2/EYFP mice (JAX B6.Cg-Tg), both male and female, of at least 3 months of age were used for the following experiments. A complete description of head-plate installation, water restriction procedure and behavioral apparatus has been described in previous work [7, 9]. Following head-plate installation, mice were housed with littermates and singly housed if fighting occurred. Mice were provided food *ad libitum*. 7 days prior to training, mice were water restricted to 1mL of water per day. During this period, a daily health and weight assessment was completed to ensure mice were healthy. All procedures were approved under USC IACUC protocols 20169 and 20731.

### METHOD DETAILS

#### Object localization task

Mice were trained in a whisker-based go/no-go localization task. Using a single whisker (C2), mice learned to identify a smooth 0.6mm diameter pole 7-12mm lateral from the whisker pad as either a posterior rewarded location (go) or anterior unrewarded location (no-go). Pole positions were presented across a continuous range of 10mm along the anteroposterior axis with a go/nogo discrimination boundary at the center of this range. The pole was positioned by a pair of stepper linear actuators with 99 nm resolution, 25 μm accuracy and < 5μm repeatability (Zaber NA11B30-T4). To avoid potential pole motion duration clues to position, between trials the motors first moved to the discrimination boundary then to the presentation location. To avoid potential ultrasonic clues associated with stepper motor function, the pole location was randomly jittered 0-127 microsteps (0-25 μm) on each trial. The pole was vertically lifted into reach by a pneumatic linear slider (Festo) which also provided a sound cue on pole presentation onset. The position of this slider and the valve, and thus the location and amplitude of this cue sound, is fixed for all trials, confirmed by audio recording with an Earthworks M50 ultrasonic microphone. Mice made their decisions by licking or withholding licking to an electrical port during stimulus presentation. 4 trial outcomes were available: hit and miss or false alarm and correct rejection by licking or not licking on a go or nogo trial. On hit trials, a water reward (4-8μL) was dispensed. The total amount of water dispensed of the session was limited only by the number of trials the mice chose to perform. False alarm trials led to a 2 s timeout that reset upon each lick. Correct rejection and miss trials were unpunished.

Each trial was 4000 ms or longer. The pole was triggered to rise at 500 ms from trial start and came into touch range within ~200 ms. The sampling period was 0-750 ms after pole onset. Licking within this time block had no effect. The answer period was 1250-2000 ms. Licking within this time block led to Hit or False Alarm outcome. Licking in this time also prolonged the period of pole presentation to provide the opportunity for additional sensory feedback to help learning. The extended presentation time does not affect any analyses since only pre-lick touches are considered in this work. The inter-trial interval was 2000 ms.

To quantify learning rates all sessions leading up to the expert session were used, excluding one to two rig acclimation sessions. Expert threshold was set at > 75% accuracy smoothing across 200 trials.

#### Training

15 mice were trained in the object localization task. In the first sessions, the farthest go position was set ~30 degrees anterior of the resting whisker position. Optimal learning was achieved by first setting a gap between go and nogo ranges and slowly reducing that gap as performance improved. The initial gap set between go and no-go ranges were 4mm. Once mice reached > 75% accuracy over 200 trials, this gap was reduced in 1mm increments till the go and nogo ranges were contiguous, with their shared border defined as the discrimination boundary.

Five expert mice in the object localization task were tested on the angle/distance task. Angles and distances were calculated from the estimated follicle position at the discrimination boundary to the full range of pole positions in the object localization task. During the angle/distance task, 120 trials of the object localization task were first presented to establish baseline performance levels. Next, angle trials or distance trials were presented at 50% chance levels for the remainder of the session.

#### Whisker motion acquisition and analysis

Whisker behavior was captured for 4 s spanning the period prior to pole onset to response window. Video frames were acquired at 1000 fps using Basler acA200-340kmNIR camera and Edmund Optics 0.18× ½” GoldTL Telecentric Lens (Model # 52-258) under 940 nm illumination on Streampix 6 software. Whisker position was tracked using Janelia Whisker Tracker (https://www.janelia.org/open-science/whisk-whisker-tracking [10]). A mask was traced from the edge of the fur and whisker follicle was estimated 1mm back from the mask. The whisker’s azimuthal angle was quantified at the point of intersection of the mask and whisker trace, to avoid tracking noise in the fur. Whisking midpoint, amplitude and phase was decomposed from this angle using the Hilbert transform. Hilbert decompositions were calculated from band-pass filtered (6-60 Hz, Butterworth) whisker angle time-series. Whisking amplitude is defined as the magnitude of the Hilbert transform of the filtered whisker angle. Whisking midpoint is defined as the filtered (6-60 Hz) difference between the raw whisker angle time-series and the band-pass filtered signal. Whisking phase is defined as the phase angles of the Hilbert transform of the filtered whisker angle time-series. Whisker curvature was measured at 3-5mm out from the mask.

The precise millisecond of touch was determined through custom MATLAB software (https://github.com/hireslab/HLab_Whiskers) using distance to pole and change in whisker curvature, followed by manual curation of images of uncertain whisker and pole intersections.

### QUANTIFICATION AND STATISTICAL ANALYSIS

In all analyses, we considered only whisker motion and touch before the decision lick, the first lick of the answer period. On trials without licking, the median decision lick time on lick trials was used as the decision point. Licks before the answer period were ignored. To minimize the effects of change internal states of motivation, attention, satiety or frustration, the set of the 200 highest performing contiguous trials in a single session per mouse was used for all analyses. Trials (0-15) where the animal was grooming or the video dropped 1 or more frames were removed from this set of 200.

#### Adaptive whisking analyses

Pre-touch windows were defined as the time from stimulus onset to first touch. Post-touch windows were set as time of first touch to the first lick. If no first touch or first lick was present, the median first touch time or median first lick time of the session was used. A whisk is defined as the number of whisking peaks with a whisking amplitude of 5 degrees or greater. The difference in distributions is quantified using Kullback-Leibler divergence from using KLDiv from Mathworks (https://www.mathworks.com/matlabcentral/fileexchange/20688-kullback-leibler-divergence).

#### Trial type and choice prediction

Retraction and protraction touches occur with ~pi radian offset in phase, which makes phase difficult to express as a linear function. Therefore we excluded retraction touches and trials with exclusively retraction touches for the Hilbert transform decoders (Figures 7C-7F). For all other analysis retraction touches were included.

- The list of features used to predict trial type (go/no-go) or choice (lick/no-lick) and their description are:
- motor position (the horizontal motor position in microsteps for each trial)
- touch presence (the presence or absence of a touch pre-decision)
- touch counts (the number of touches pre-decision)
- roll angle (the mean whisker curvature 1 ms prior to touch for each trial)
- whisk latency (the mean time in milliseconds from the nearest whisking trough prior to touch for each trial)
- cue latency (the time of first touch from cue onset in milliseconds)
- radial distance (the mean radial distance from follicle at touch to pole position for each trial)
- angle (the mean whisker angle at touch for each trial)
- phase (the mean phase of the whisker at touch for each trial)
- amplitude (the mean amplitude of the whisker at touch for each trial)
- midpoint (the mean midpoint of the whisker at touch for each trial)
- combined (curvature, cue latency, whisk latency, touch counts, radial distance and angle for each trial)
- hilbert decomposition (phase, amplitude, and midpoint)
- For features using multiple predictors, each feature was mean normalized using the following equation:

$$x^{\prime }=\frac{x-\text{mean}(x)}{\max(x)-\min(x)}$$

The logistic classifier was adapted from Andrew Ng’s Machine Learning Course (https://www.coursera.org/learn/machine-learning) and modified to include lasso regularization.

Sigmoid link function:

$$h_{\theta }(x)=g(\theta^{⊺}x)$$

$$\text{where}\;g(z)=\frac{1}{1+e^{-z}}$$

Cost function:

Cost(hθ(x),y)={−log(hθ(x))ify=1−log(1−hθ(x))ify=0}

$$J(\theta )=\frac{1}{m}\sum_{i=1}^{m}[-y^{i}\;\log(h_{\theta }(x^{i}))-(1-y^{i})\log(1-h_{\theta }(x^{i}))]+\mathrm{Regularization}$$

L1 lasso regularization equation:

$$\lambda *\sum_{i=1}^{N}|\theta_{i}|$$

Where *λ* is the regularization parameter, *θ* are the partial regression coefficients of the model and N is the number of parameters.

Gradient (partial derivative of the cost function):

$$\frac{\partial J(\theta )}{\partial \theta_{j}}=\frac{1}{m}\sum_{i=1}^{m}(h_{\theta }(x^{i})-y^{i})x_{j}^{\left(i\right)}$$

The cost function was minimized through the fmincg MATLAB function. The inputs of this function are the cost and the gradient:

$$J(\theta )\;\text{and}\;.\frac{\partial J(\theta )}{\partial \theta_{j}}$$

#### Classifier model evaluation

For each set of features the optimal regularization parameter *λ*, classifier performance and partial regression coefficients *θ* were evaluated across 20 iterations with 5-fold stratified cross-validation. Optimal *λ* was chosen as the mean *λ* value between the *λ* that yielded the lowest error and the first *λ* that yielded error one SEM away from the minimum.

Classifier performance was calculated using Matthew’s correlation coefficient (MCC). MCC provides an unbiased metric of model performance in light of imbalanced datasets [57]. MCC values range from 1 to −1 with 1 meaning perfect model performance, 0 meaning chance, and −1 meaning all predictions are errors. The MCC was calculated using the following equation:

$$\mathrm{MCC}=\frac{\mathrm{TP}*\mathrm{FN}-\mathrm{FP}*\mathrm{FN}}{\sqrt{\left(\mathrm{TP}+\mathrm{FP})*(\mathrm{TP}+\mathrm{FN})*(\mathrm{TN}+\mathrm{FP})*(\mathrm{TN}+\mathrm{FN}\right)}}$$

where *TP* are true positives, *TN* are true negatives, *FN* are false negatives and *FP* are false positives predictions.

In order to interpret the weight of the logistic classifier, partial regression coefficients were converted to odds ratios using the following equation:

$$\mathrm{Odds}\;\mathrm{Ratio}=e^{\theta }$$

Odds ratios were normalized between 0 and 1 and multiplied by their respective sign for each cross-validation step and averaged to calculate the normalized weight of each feature in prediction.

### DATA AND CODE AVAILABILITY

The datasets generated during this study are available on the Hires Lab Dropbox repository at (https://www.dropbox.com/sh/bjla01r0bzt49j7/AAAzMjaq2mZSH5Gp8sf_UY5ga).

The code generated during this study and any updated links to the datasets are available on GitHub at (https://github.com/hireslab/Pub_LocalizationBehavior).

## Supplementary Material

1

## Figures and Tables

**Table:** KEY RESOURCES TABLE

REAGENT or RESOURCE	SOURCE	IDENTIFIER
Experimental Models: Organisms/Strains		
Mouse: B6.Cg-Tg(Slc32a1-COP4*H134R/EYFP)8Gfng/J	The Jackson Laboratory	JAX: 014548
Software and Algorithms		
MATLAB v.2013b and v.2018b	MathWorks	2013b and 2018b
Ephus	Vidrio Technologies	http://www.scanimage.vidriotechnologies.com/display/ephus/Ephus
StreamPix	Norpix	https://www.norpix.com/news/newsletters/streampix6.php
Janelia Whisker Tracker	[10]	https://www.janelia.org/open-science/whisk-whisker-tracking
HLab_Whiskers package	Hires Lab	https://github.com/hireslab/HLab_Whiskers
Pub_LocalizationBehavior package	This paper	https://github.com/hireslab/Pub_LocalizationBehavior
Localization behavior dataset	This paper	https://www.dropbox.com/sh/bjla01r0bzt49j7/AAAzMjaq2mZSH5Gp8sf_UY5ga
Other		
High speed CMOS Camera	Basler	acA200-340kmNIR
Telecentric lens	Edmund Optics	0.18× ½" GoldTL™ Telecentric Lens (Model # 52-258)

## Highlights

- Mice discriminate anteroposterior object locations to ≤ 0.5 mm using a single whisker
- Mice locate objects using targeted, noisy exploration that is adaptive to touch
- Mice don’t use roll angle, precise timing, or distance to locate these objects
- Whisking midpoint and the number of touches made best explains localization acuity
