# Supplementary material for: The Sensorimotor Basis of Whisker-Guided Anteroposterior Object Localization in Head-Fixed Mice
Source: Curr Biol. Author manuscript; Available in PMC 2019 Oct 1. (PMC6771421; doi:10.1016/j.cub.2019.07.068)
Supplement: 1 [file NIHMS1049141-supplement-1.pdf]

Current Biology, Volume *29*

Supplemental Information

The Sensorimotor Basis of Whisker-Guided Anteroposterior Object Localization in Head-Fixed Mice

Jonathan Cheung, Phillip Maire, Jinho Kim, Jonathan Sy, and Samuel Andrew Hires

A

Model MCC in predicting trial type comparison

Feature	Feature	p-value	sig.
Roll angle	Cue latency	0.99	0
Roll angle	Whisk latency	0.97	0
Roll angle	Touch count	6.0e-4	1
Roll angle	Radial distance	2.0e-4	1
Roll angle	Angle	1.0e-4	1
Cue latency	Whisk latency	0.76	0
Cue latency	Touch count	1.0e-4	1
Cue latency	Radial distance	1.0e-4	1
Cue latency	Angle	1.0e-4	1
Whisk latency	Touch count	7.0e-4	1
Whisk latency	Radial distance	3.0e-3	1
Whisk latency	Angle	6.0e-4	1
Touch count	Radial distance	0.99	0
Touch count	Angle	1.0	0
Radial distance	Angle	1.0	0

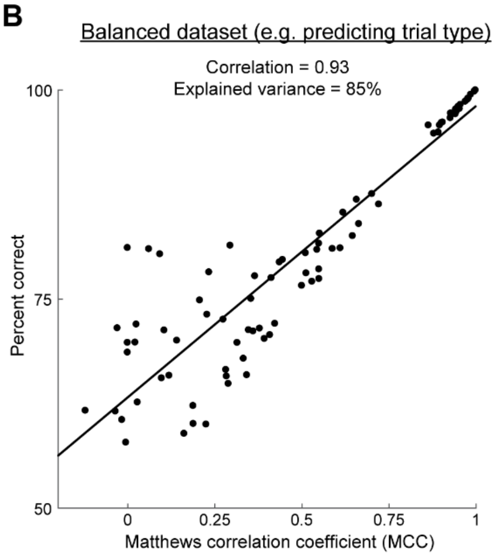

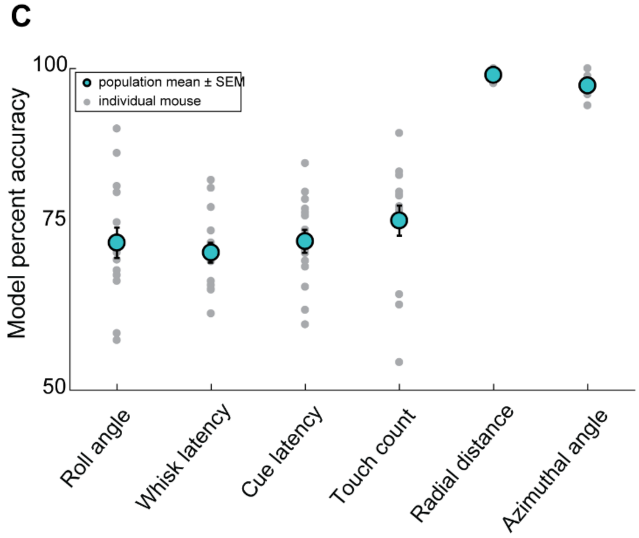

Figure S1. MCC versus accuracy for the trial type prediction models, Related to Figure 4. A) P-values comparison between the trial type predictive accuracy of pairs of features using a 1-way ANOVA. B) In a balanced distribution of predicted values (e.g. trial type), both accuracy and MCC values provide complementary information of model performance. (r = 0.93, R^2 = 0.85). C) Model accuracy reported instead of MCC for Fig 4G.

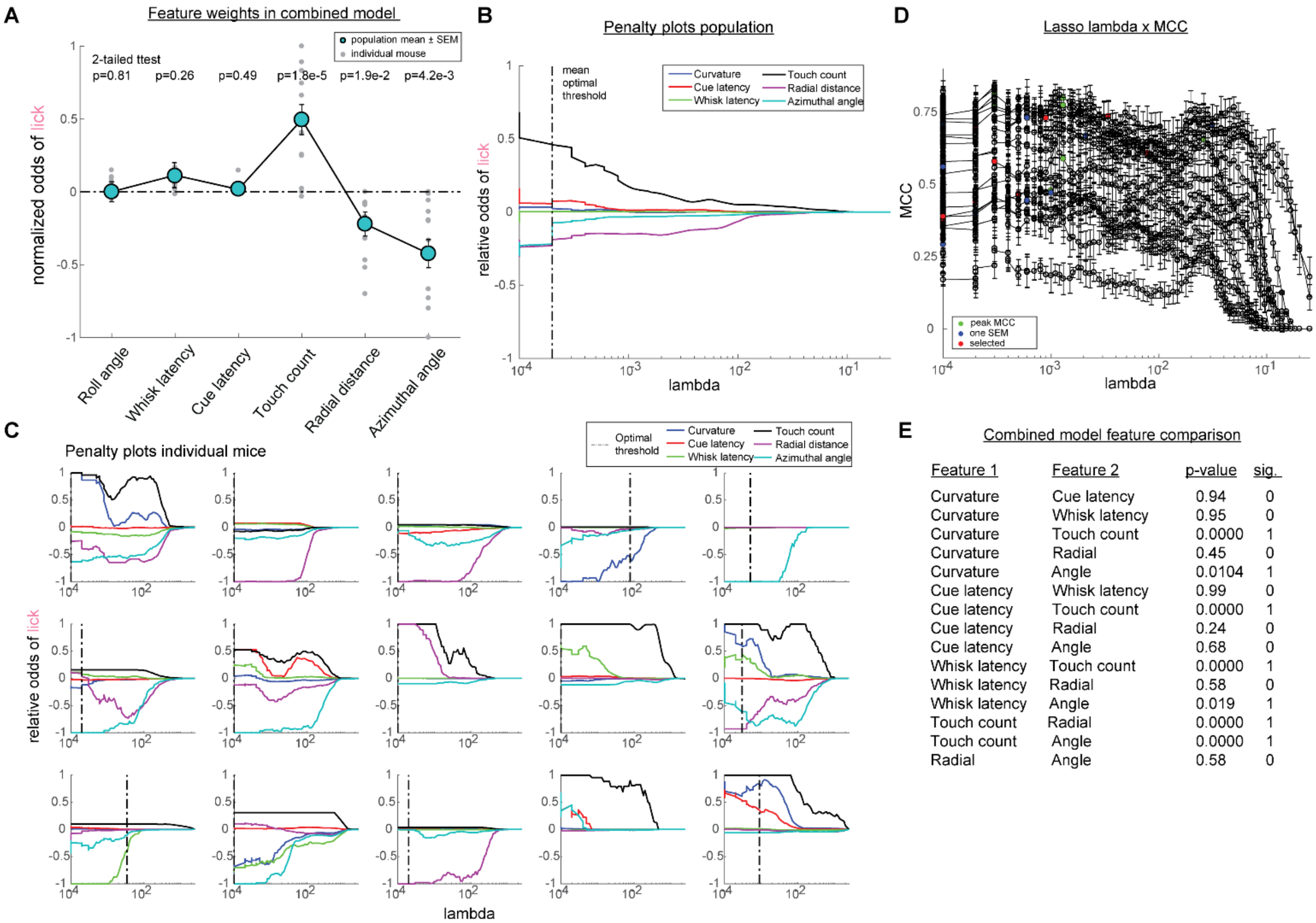

Feature 1	Feature 2	p-value	sig.
Curvature	Cue latency	0.94	0
Curvature	Whisk latency	0.95	0
Curvature	Touch count	0.0000	1
Curvature	Radial	0.45	0
Curvature	Angle	0.0104	1
Cue latency	Whisk latency	0.99	0
Cue latency	Touch count	0.0000	1
Cue latency	Radial	0.24	0
Cue latency	Angle	0.68	0
Whisk latency	Touch count	0.0000	1
Whisk latency	Radial	0.58	0
Whisk latency	Angle	0.019	1
Touch count	Radial	0.0000	1
Touch count	Angle	0.0000	1
Radial	Angle	0.58	0

Figure S2. Combined model weights and lasso feature reduction in choice prediction, Related to Figure 4. A) Comparison of normalized odds for predicting choice in the combined model. Gray dots denote the weights for each mouse and turquoise dots denote the mean ± SEM across the population. B) Penalty plots for the population mean. C) MCC performance as a function of lambda. Green dots denote peak MCC value, blue dots denote MCC values 1 SEM away from peak, and red dot denotes mean lambda of peak and 1 SEM away (i.e. chosen optimal lambda). D) Penalty plots for individual mice. E) p-values comparing classifier weights; one-way ANOVA.

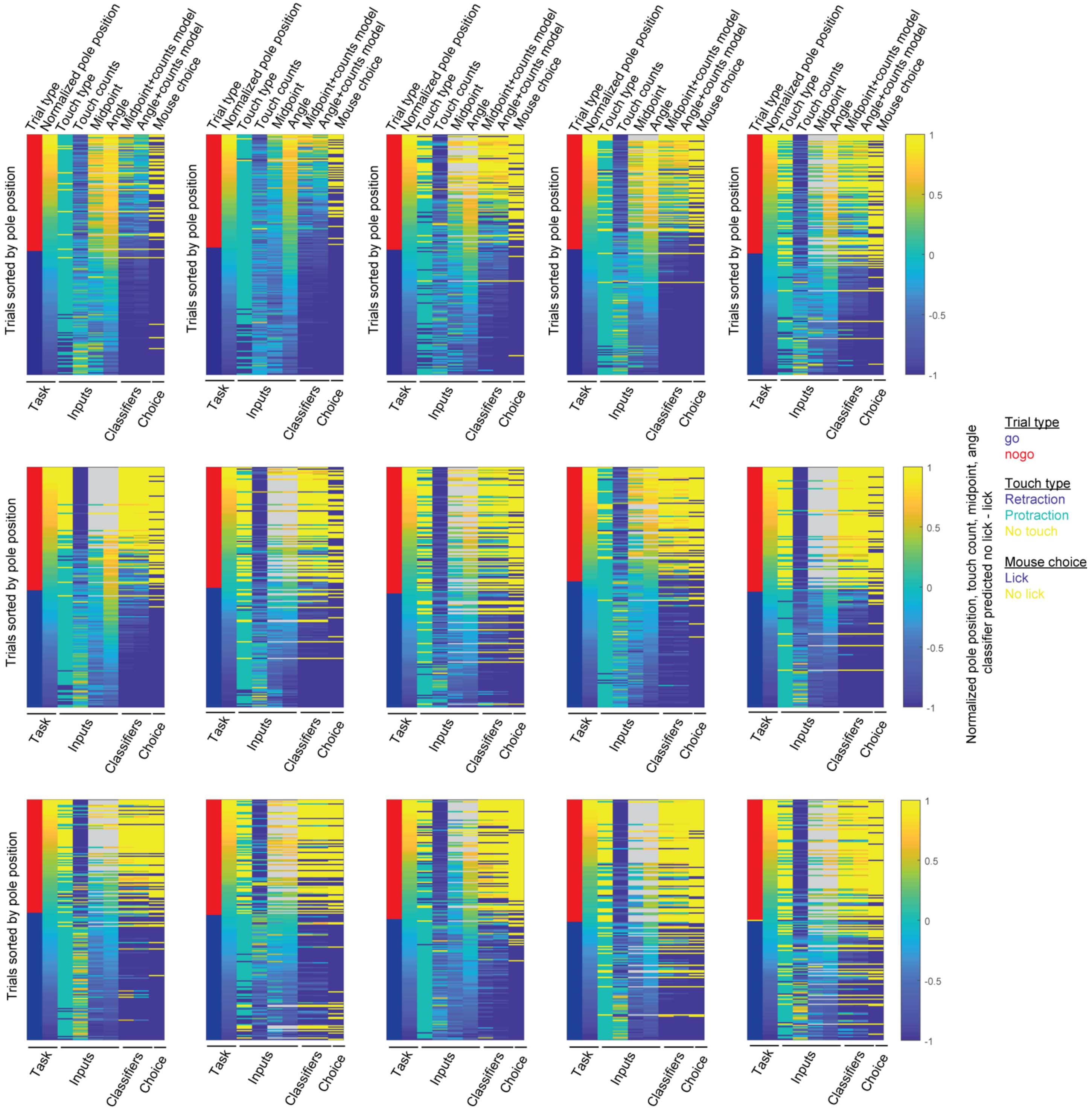

Figure S3. Heatmap of model comparisons as in Figure 7G for each mouse, Related to Figure 7.

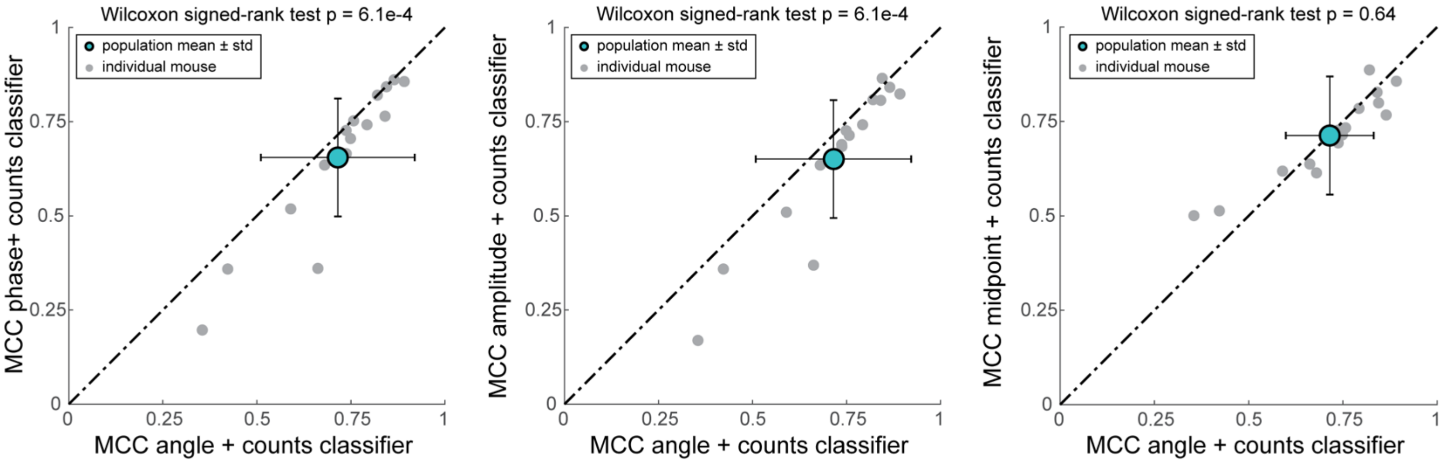

Figure S4. Comparison of touch count + touch angle classifier performance versus touch count + each Hilbert component individually, Related to Figure 7.

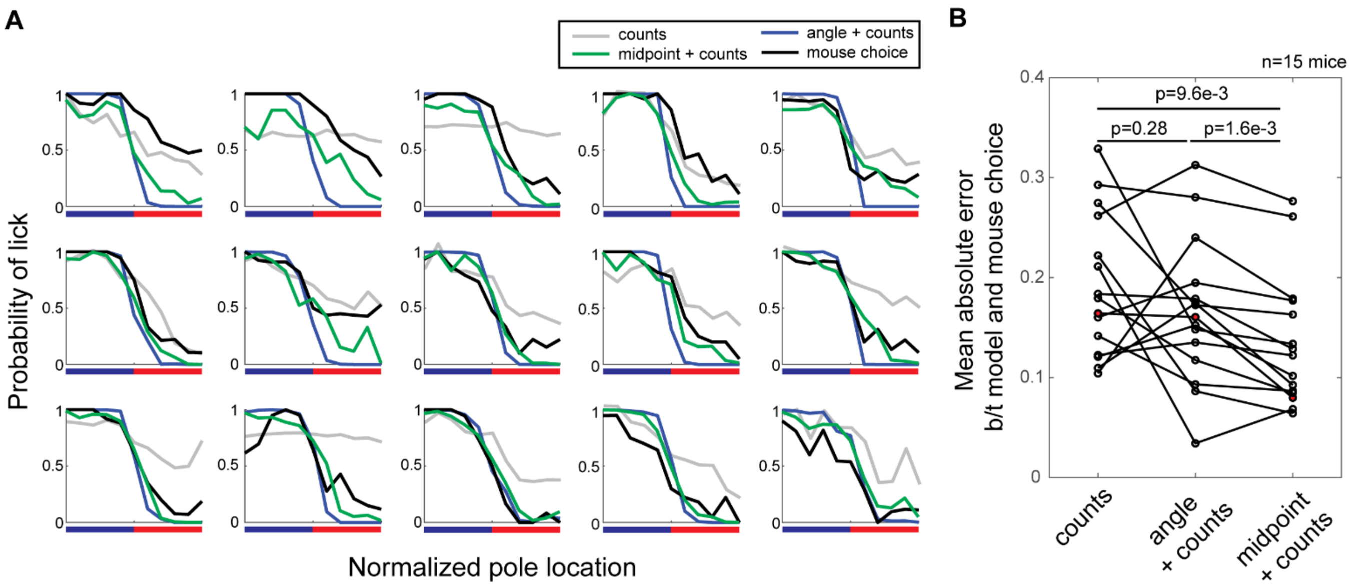

Figure S5. Comparison of models for optimal trial type discrimination and mouse behavior, Related to Figure 7. A) Psychometric curves for optimal trial type discrimination performance using counts, midpoint+counts and angle+counts compared against mouse choice. B) Comparison of mean absolute error between models and mouse choice for counts (0.19±0.07std), angle+counts(0.17±0.07std) and midpoint+counts (0.13±0.06std) classifiers (paired t-test). Red filled dot denotes example in Figure 7I.
